# Increasing angiotensin-converting enzyme concentrations and absent angiotensin-converting enzyme activity are associated with adverse kidney outcomes in pediatric septic shock

**DOI:** 10.1186/s13054-023-04518-2

**Published:** 2023-06-12

**Authors:** Naomi Pode-Shakked, Giovanni Ceschia, James E. Rose, Stuart L. Goldstein, Natalja L. Stanski, the Genomics of Pediatric Septic Shock Investigators

**Affiliations:** 1 Cincinnati Children’s Hospital Medical Center, 3333 Burnet Ave, Cincinnati, OH 45208 USA; 2 Sackler Faculty of Medicine, Tel-Aviv University, P.O. Box 39040, 6997801 Tel Aviv, Israel; 3 Pediatric Nephrology Unit, Department of Women’s and Children’s Health, University-Hospital of Padova, Via Giustiniani 3, 35128 Padua, Italy; 4 Department of Pediatrics, University of Cincinnati College of Medicine, 3230 Eden Ave, Cincinnati, OH 45267 USA

**Keywords:** Sepsis, Shock, Pediatrics, Acute kidney injury, Renin–angiotensin–aldosterone system, Angiotensin-converting enzyme, Renin, Angiotensin II, Biomarkers

## Abstract

**Background:**

Sepsis-induced endothelial dysfunction is proposed to cause angiotensin-converting enzyme (ACE) dysfunction and renin–angiotensin–aldosterone system (RAAS) derangement, exacerbating vasodilatory shock and acute kidney injury (AKI). Few studies test this hypothesis directly, including none in children. We measured serum ACE concentrations and activity, and assessed their association with adverse kidney outcomes in pediatric septic shock.

**Methods:**

A pilot study of 72 subjects aged 1 week–18 years from an existing multicenter, observational study. Serum ACE concentrations and activity were measured on Day 1; renin + prorenin concentrations were available from a previous study. The associations between individual RAAS components and a composite outcome (Day 1–7 severe persistent AKI, kidney replacement therapy use, or mortality) were assessed.

**Results:**

50/72 subjects (69%) had undetectable ACE activity (< 2.41 U/L) on Day 1 and 27/72 (38%) developed the composite outcome. Subjects with undetectable ACE activity had higher Day 1 renin + prorenin compared to those with activity (4533 vs. 2227 pg/ml, *p* = 0.017); ACE concentrations were no different between groups. Children with the composite outcome more commonly had undetectable ACE activity (85% vs. 65%, *p* = 0.025), and had higher Day 1 renin + prorenin (16,774 pg/ml vs. 3037 pg/ml, *p* < 0.001) and ACE concentrations (149 vs. 96 pg/ml, *p* = 0.019). On multivariable regression, increasing ACE concentrations (aOR 1.01, 95%CI 1.002–1.03, *p* = 0.015) and undetectable ACE activity (aOR 6.6, 95%CI 1.2–36.1, *p* = 0.031) retained associations with the composite outcome.

**Conclusions:**

ACE activity is diminished in pediatric septic shock, appears uncoupled from ACE concentrations, and is associated with adverse kidney outcomes. Further study is needed to validate these findings in larger cohorts.

**Supplementary Information:**

The online version contains supplementary material available at 10.1186/s13054-023-04518-2.

## Introduction:

Septic shock is common in the pediatric intensive care unit and associated with increased morbidity and mortality [1, 2]. Children with septic shock often develop acute kidney injury (AKI), which confers increased risk of poor outcomes [3]. Unfortunately, the treatment of septic shock and sepsis-associated AKI remains limited to supportive care, as no specific disease-modifying therapies exist [4]. A better understanding of the mechanisms of organ dysfunction in septic shock is necessary to improve outcomes.

**Fig. 1:**
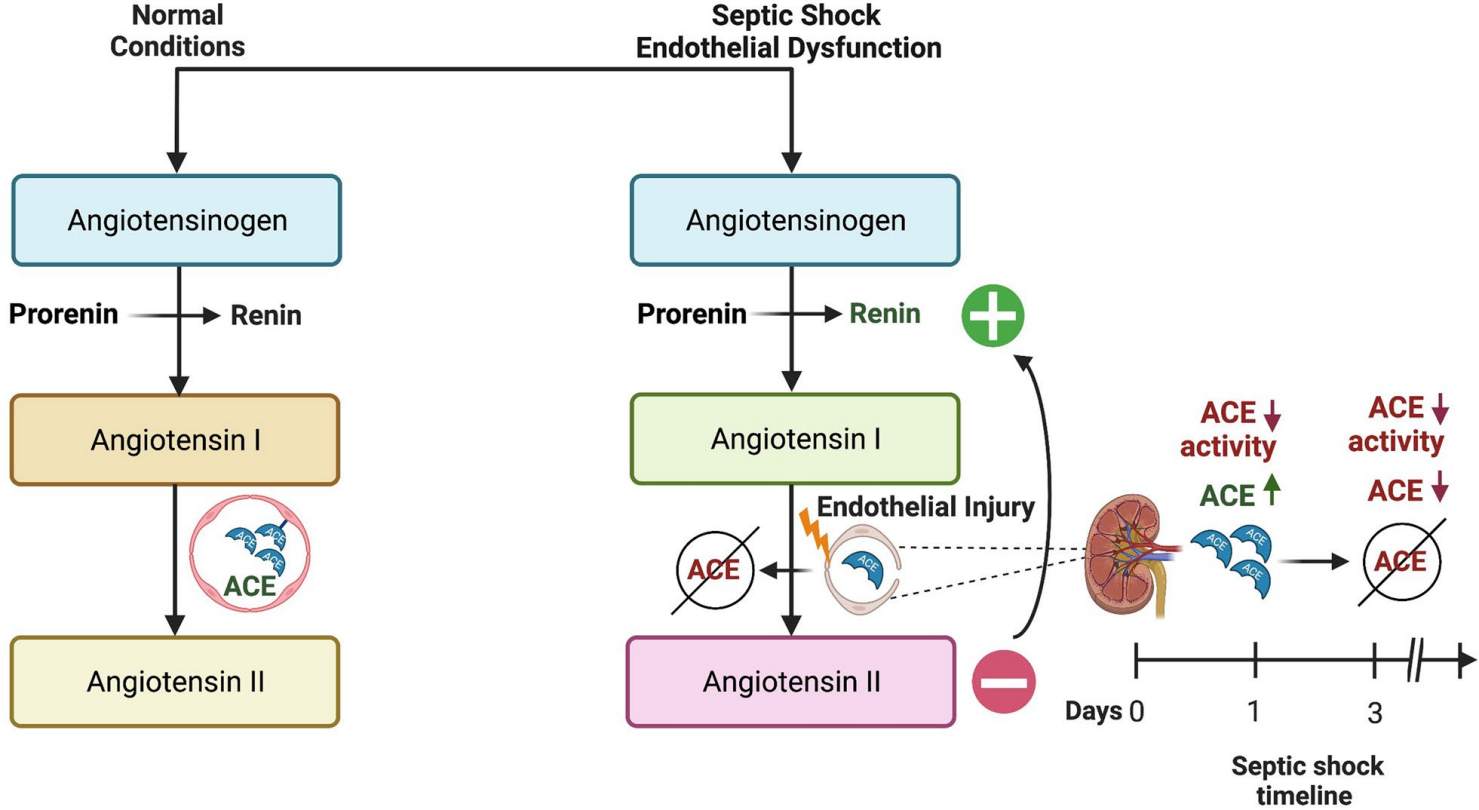
Proposed Mechanism of Renin-Angiotensin System Derangement in Septic Shock. Under normal circumstances, inactive prorenin undergoes proteolytic activation to renin in the kidney, where active renin is stored and released immediately upon stimulation of the juxtaglomerular apparatus. In the setting of septic shock and associated endothelial damage, angiotensin-converting enzyme (ACE) function is proposed to be impaired, resulting in acutely decreased production of angiotensin II and resultant increase in serum renin concentrations secondary to release of active enzyme. While proteolytic activation of prorenin to renin is also ongoing, this process is influenced more by chronic stimuli as opposed to acute stimuli. Finally, the right lower panel demonstrates a putative etiology for the uncoupling of ACE activity and ACE levels at early stages of pediatric septic shock; at Day 1 following endothelial injury, ACE stored in kidney endothelia is released to the circulation, possibly resulting in transient increase of ACE levels with subsequent decrease when storage is exhausted

There is increasing evidence that the renin–angiotensin–aldosterone system (RAAS) is dysregulated in critical illness [5–9], and may represent a modifiable target for treatment of patients with septic shock and AKI [6, 9, 10]. Specifically, it is postulated that endothelial injury results in ACE dysfunction with subsequent decreased conversion of angiotensin I (AngI) to angiotensin II (AngII) (a potent vasoconstrictor), exacerbating vasodilatory shock (Fig. 1) [6, 9]. AngII also has direct effects on intrarenal circulation, causing preferential vasoconstriction of efferent arterioles to increase glomerular perfusion pressure, and thus decreased levels may also exacerbate AKI [10]. This hypothesis is supported by evidence demonstrating increased AngI/AngII ratios in adults with catecholamine resistant vasodilatory shock (CRVS) [6, 9], however, few studies have directly measured ACE concentrations [11] and none have measured ACE activity. The importance of testing this hypothesis directly is underscored by the availability of synthetic AngII for treatment of CRVS.

We performed a pilot study measuring serum ACE concentrations and activity in a cohort of children with septic shock. We hypothesized children with septic shock would have low ACE concentrations and activity, and that these values would be associated with increased renin concentrations and adverse kidney outcomes.

## Methods

Further details are provided in the Additional file 2.

### Study design and patient selection

We performed an exploratory analysis of subjects from a multicenter observational study of children with septic shock. The original study protocol, which has been published in detail [12], was approved by local Institutional Review Boards prior to enrollment. From an existing secondary analysis cohort examining serum renin + prorenin concentrations (n = 233) [13], 72 subjects were randomly selected for inclusion in our study based on residual serum availability, and ensuring representative incidences of severe persistent AKI, kidney replacement therapy (KRT) use, and mortality (Additional file 1: Fig. S1).

### Measurements

Day 1 serum was analyzed for ACE concentrations using a human ACE Quantikine ELISA Kit (R&D Systems Inc., Minneapolis, MN, USA) and activity using a high sensitivity enzymatic assay (Bulhmann Diagnostics Corp, Amherst, NH, USA). Renin + prorenin concentrations were measured as part of a previous study [13]. In children, a normal ACE concentration is ~ 200 ng/ml [14], and ACE activity is expected to be 20–50% higher than adults (16–85 U/L) [15, 16]. A normal serum renin is 2–59 pg/ml [6]. All other clinical/laboratory data were measured as part of clinical care.

### Outcomes and definitions

We assessed the values of the individual RAAS components and their associations with a composite outcome: Day 1–7 severe persistent AKI (Kidney Disease Improving Global Outcomes Stage 2 or higher lasting by serum creatinine [SCr] for ≥ 48 h [17]), and/or Day 1–7 KRT use, and/or 28-day mortality. Baseline SCr values were unknown, and were imputed using calculated body surface area (m^2^) and an eGFR of 120 ml/min per 1.73 m^2^ [18]. Additional secondary outcomes are outlined in the Additional file 2.

### Statistical analysis

Data were described using medians, interquartile ranges, frequencies and percentages. Comparisons between groups were performed using Wilcoxon rank-sum or Chi-square test. ACE and renin + prorenin concentrations were treated as continuous variables; ACE activity was treated as a dichotomous variable (detectable: > 2.41 U/L or undetectable: < 2.41 U/L). Multivariable logistic regression was utilized to assess for associations between RAAS components and the composite outcome, after adjustment for illness severity by PRISM III score and vasoactive burden by vasoactive-inotropic score (VIS). A *p*-value of < 0.05 was considered statistically significant. Analyses were performed using Sigmaplot 14.0 (Systat Software Inc., San Jose, CA, USA).

## Results

### ACE activity is low in children with septic shock

Fifty of 72 subjects (69%) had undetectable ACE activity (< 2.41 U/L) on Day 1. When compared to subjects with detectable activity, those without had higher PRISM III scores; there were no other demographic differences (Table 1). Subjects with undetectable ACE activity had higher Day 1 renin + prorenin concentrations (4533 vs. 2227 pg/ml, *p* = 0.017). There was no difference in ACE concentration between those with and without detectable ACE activity (122 vs. 102 ng/ml, *p* = 0.094).

**Table 1:** Demographic, clinical and outcome data by patients by the presence or absence of detectable (> 2.41 U/L) angiotensin-converting enzyme activity and for patients with angiotensin converting enzyme concentrations above and below the cohort median

	All	ACE activity < 2.41 U/L	ACE activity > 2.41 U/L	Comparison	ACE level < 104 ng/ml	ACE level > 104 ng/ml	Comparison
N (% cohort)	72	50 (69)	22 (31)	–	36 (50)	36 (50)	–
Age, years	12.1 (3.2, 17.7)	12.1 (4.1, 7.6)	13.1 (0.9, 20.4)	0.91	12.7 (3.6, 17.9)	11 (2.3, 17.1)	0.67
Sex, male (%)	27 (38)	16 (32)	11 (50)	0.15	16 (44)	11 (31)	0.22
PRISM III	11.5 (8.3, 15)	13 (9.8, 16)	10 (6.5, 12)	0.012	10.7 (8.3, 15)	12 (8.3, 15.8)	0.86
D1 Vasoactives, yes (%)	63 (88)	44 (88)	19 (86)	1.0	32 (89)	31 (86)	1.0
Epinephrine (low)	5 (7.9)	3 (6.8)	2 (10.5)	0.17	4 (12.5)	1 (3.2)	0.57
Epinephrine (high)	11 (17.5)	10 (22.7)	1 (5.3)		6 (18.8)	5 (16.1)	
Norepinephrine	25 (39.7)	19 (43.2)	6 (31.5)		12 (37.5)	13 (42)	
Combination	18 (28.6)	9 (20.5)	9 (47.4)		9 (28.1)	9 (29)	
Other	4 (6.3)	3 (6.8)	1 (5.3)		1 (3.1)	3 (9.7)	
D1 VIS	12.5 (5, 30)	11.5 (5, 30)	12.5 (5, 36)	0.82	10 (5, 25)	18.6 (8.3, 45)	0.11
D1 MV, yes (%)	48 (67)	35 (70)	13 (59)	0.37	25 (69)	23 (64)	0.62
Organism, n (%)				0.92			0.40
Gram positive	13 (18.1)	9 (18)	4 (18.2)		4 (11.1)	9 (25)	
Gram negative	19 (26.4)	13 (26)	6 (27.3)		12 (33.3)	7 (19.4)	
Viral	2 (2.8)	1 (2)	1 (4.5)		1 (2.8)	1 (2.8)	
Fungal	6 (8.3)	5 (10)	1 (4.5)		4 (11.1)	2 (5.6)	
None	32 (44.4)	22 (44)	10 (45.5)		15 (41.7)	17 (47.2)	
D1 Renin + Prorenin, pg/ml	3940 (1749, 11,671)	4533 (2782, 21,834)	2227 (1053, 6284)	0.017	3288 (1267, 7557)	6096 (2539, 19,903)	0.092
D1 ACE, ng/ml	104 (78, 149)	102 (71, 144)	122 (92, 166)	0.094	**–**	**–**	**–**
D1-7 Severe Persistent AKI, n (%)	18 (25)	15 (30)	3 (14)	OR 2.7 (95% CI 0.70–10.6, *p* = 0.14)	3 (8)	15 (42)	OR 7.9 (95%CI 2.0–30.5, *p* = 0.001)
D1-7 KRT, n (%)	12 (17)	10 (20)	2 (9)	OR 2.5 (95% CI 0.50–12.5, *p* = 0.32)	2 (6)	10 (28)	OR 6.5 (95%CI 1.3–32.4, *p* = 0.011)
28-day PICU-Free Days	13.5 (0, 24)	13 (0, 24)	20.5 (0.75, 25)	0.27	17.5 (0, 24)	12 (0, 24)	0.36
7-day Vasoactive-Free Days	4 (2, 5)	4 (1.8, 5)	4.5 (2.75, 6)	0.40	4 (3.25, 5)	3.5 (1, 5)	0.17
28-day Mortality, n (%)	19 (26)	16 (32)	3 (14)	OR 3.0 (95%CI 0.77–11.6, *p* = 0.10)	7 (19)	12 (33)	OR 2.1 (95%CI 0.71–6.1, *p* = 0.18)
Composite Outcome	27 (38)	23 (46)	4 (18)	OR 3.8 (95%CI 1.1–13.0, *p* = 0.025)	9 (25)	18 (50)	OR 3.0 (95%CI 1.1–8.1, *p* = 0.028)

Continuous data reported as median (IQR)

PRISM III, Pediatric Risk of Mortality Score III; D, day; Low dose epinephrine, < 0.1 mcg/kg/min; High dose epinephrine, ≥ 0.1 mcg/kg/min; Combination, epinephrine ± vasopressin ± norepinephrine; Other, dopamine, vasopressin or milrinone monotherapy; VIS, vasoactive-inotrope score; MV, mechanical ventilation; ACE, angiotensin-converting enzyme; AKI, acute kidney injury; KRT, kidney replacement therapy; Composite Outcome, Day 1–7 severe persistent AKI, Day 1–7 KRT, and/or 28-day mortality

### RAAS derangement is associated with adverse kidney outcomes

Among 72 subjects, 27 (38%) developed the composite outcome (Table 2). There were no demographic differences between those who developed the composite outcome and those who did not; however, those with the composite outcome had higher VIS (Table 2). Additionally, undetectable ACE activity occurred more frequently (85% vs. 60%, *p* = 0.025), and Day 1 renin + prorenin (16,774 pg/ml vs. 3037 pg/ml, *p* < 0.001) and ACE concentrations (149 vs. 96 pg/ml, *p* = 0.019) were higher in children with the composite outcome.

**Table 2:** Bivariate analysis and multivariable logistic regression analysis examining the associations with the composite outcome of Day 1–7 severe persistent acute kidney injury, Day 1–7 kidney replacement therapy use, and/or 28-day mortality

Variable	Bivariate analysis			Multivariable logistic regression	
	Composite outcome	No composite outcome	p-value	Adjusted odds ratio (95% CI)	p-value
N (%) cohort	27 (38)	45 (62)	–	–	–
Age, years	6.1 (2.5, 20.2)	14 (4.8, 17.2)	0.45	–	–
Sex, male (%)	10 (37)	17 (38)	0.95	–	–
PRISM III	13 (9, 19)	10.4 (8, 13.5)	0.071*	1.09 (0.98–1.2)	0.11
D1 VIS	25 (10, 44)	10 (2, 23.5)	0.004*	1.03 (0.99–1.07)	0.10
D1 ACE Level, ng/ml	149 (70, 177)	96 (78, 126)	0.019*	1.01 (1.02–1.03)	0.025
D1 ACE activity (U/L)					
Undetectable, n (%)	23 (85)	27 (60)	0.025*	6.6 (1.2–36.1)	0.031
D1 Renin + Prorenin, pg/ml	16,774 (3484, 28,016)	3037 (1231, 5111)	< 0.001*	1.0 (1.0–1.0)	0.07

Continuous data reported as median (IQR)

*PRISM III* Pediatric Risk of Mortality Score III, *D* day, *VIS* vasoactive-inotrope score, *ACE* angiotensin-converting enzyme

*Variables with alpha level < 0.15 on bivariate analysis were included in the

multivariate logistic regression model

**Fig. 2:**
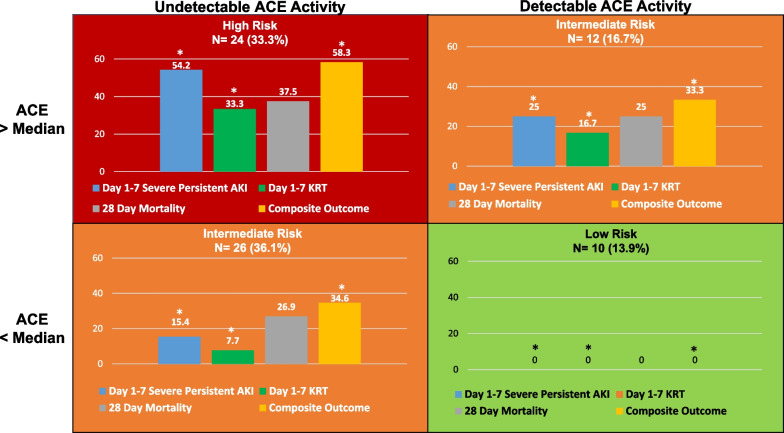
The incidence of adverse kidney related outcomes of interest by unique ACE activity and ACE concentration-derived phenotype. Four unique phenotypes were derived by the presence or absence of detectable (> 2.41 U/L) ACE activity and ACE concentration above or below the cohort median (104 ng/ml). Statistically significant comparisons (*p* < 0.05) are denoted by *

On multivariate regression (Table 2), increasing ACE concentrations (aOR 1.01, 95%CI 1.002–1.03, *p* = 0.015) and undetectable ACE activity (aOR 6.6, 95%CI 1.2–36.1, *p* = 0.031) retained associations with the composite outcome. ACE concentration above the median (104 ng/ml) was also associated with greater odds of severe persistent AKI (OR 7.9, 95%CI 2.0–31, *p* = 0.001) and KRT use (OR 6.5, 95%CI 1.3–32, *p* = 0.011) (Table 1). To better visualize the interplay between ACE concentrations and activity, four phenotypes were derived for comparison: (1) absent ACE activity/ACE above median (n = 24), (2) absent ACE activity/ACE below median (n = 26), (3) detectable ACE activity/ACE above median (n = 12), and (4) detectable ACE activity/ACE below median (n = 10) (Fig. 2, Additional file 1: Table S1). Notably, subjects with absent ACE activity/ACE above the median (Fig. 2, upper left) had uniformly worse outcomes, while those with detectable ACE activity/ACE below the median (Fig. 2**, **bottom right) suffered no adverse outcomes.

## Discussion

We report findings from a pilot study demonstrating serum ACE activity is low in children with septic shock, with over two-thirds having undetectable activity. Undetectable ACE activity was associated with upstream elevation in renin + prorenin, and with the composite outcome of severe persistent AKI, KRT use or 28-day mortality. Interestingly, though ACE concentrations were lower than expected in healthy children, *increasing* ACE concentrations were associated with poor outcomes, suggesting an uncoupling of ACE concentration and activity in this cohort.

The finding of diminished ACE activity in children with septic shock provides direct evidence in support of the hypothesis that ACE dysfunction occurs in these patients, presumably secondary to endothelial injury (Fig. 1) [6, 9]. While it is proposed that this dysfunction leads to reduced AngII production and exacerbation of vasodilatory shock and AKI, we were unable to measure AngI/AngII concentrations, which is a limitation of this study. Nevertheless, the association of absent ACE activity with poor outcomes is consistent with adult literature, which has shown similar associations in patients with CRVS, elevated renin concentrations (a proposed upstream surrogate for AngII deficiency that is more easily measured), and increased AngI/AngII ratios [6, 10]. Importantly, in these studies, investigators demonstrated that use of synthetic angiotensin II improved outcomes in patients with presumed AngII deficiency by elevated renin concentrations, including improved renal recovery [6, 10]. These data, combined with our recent data demonstrating extreme elevation of renin + prorenin concentrations in pediatric septic shock [13], suggest that further study is warranted to elucidate if AngII may be particularly beneficial in this population.

While the median ACE concentration for the cohort was lower than expected in healthy children, the finding that *higher* concentrations were associated with poor outcomes is unexpected. In this cohort, having an ACE concentration above the median was associated with severe persistent AKI and KRT use. This is contrary to a study in adults with sepsis, which demonstrated that *lower* ACE concentrations on Day 1 were associated with worse outcomes [11]. However, these values continued to decrease over the first 72 h [11], and thus we postulate that extensive endothelial injury may result in release of ACE reservoirs early in sepsis, with subsequent exhaustion over time (Fig. 1). Accordingly, data limited to Day 1 would prevent us from seeing this expected decrease in ACE concentrations. Additionally, it is possible that ACE activity and ACE levels may be uncoupled in this population, secondary to an intrinsic defect in ACE function [19] or circulating ACE inhibitors [20]. Further study is needed to examine these hypotheses.

Our study has several limitations. First, our sample size is small, and these findings need to be corroborated in a larger cohort. This was a secondary analysis of an existing study not designed to examine our hypotheses, introducing the possibility of bias. The type of shock at presentation (i.e., cold versus warm) was unknown, though 54/63 (86%) who required vasoactives received agents suggesting vasodilatory shock (high dose epinephrine, norepinephrine and/or vasopressin). We were unable to trend these concentrations over time and with changes in hemodynamics, an important limitation given the dynamic nature of critical illness. Finally, the lack of direct renin and AngI/AngII data makes direct comparison to the existing adult literature challenging. These issues should be addressed in future prospective studies.

## Conclusions

We demonstrate ACE activity is low in children with septic shock, apparently uncoupled from ACE concentrations, and associated with poor outcomes. If validated in a larger cohort, these data provide clarity on the potentially modifiable mechanisms of RAAS derangement in these patients.

## Supplementary Information

**Additional file 1**: Figures and Tables.

**Additional file 2**: Methods.

## Data Availability

The datasets used and/or analyzed during the current study are available from the corresponding author on reasonable request.

## Abbreviations

PICU: Pediatric intensive care unit
AKI: Acute kidney injury
RAAS: Renin–angiotensin–aldosterone system
ACE: Angiotensin-converting enzyme
