# Supplementary material for: Increasing angiotensin-converting enzyme concentrations and absent angiotensin-converting enzyme activity are associated with adverse kidney outcomes in pediatric septic shock
Source: Crit Care. 2023 Jun 12;27:230. doi: 10.1186/s13054-023-04518-2 (PMC10259008; doi:10.1186/s13054-023-04518-2)
Supplement: Supplementary file 1 — Additional file 1: Figures and Tables. [file 13054_2023_4518_MOESM1_ESM.docx]

**SUPPLEMENTAL TABLES AND FIGURES:**

**Figure S1: CONSORT Flow Diagram.**


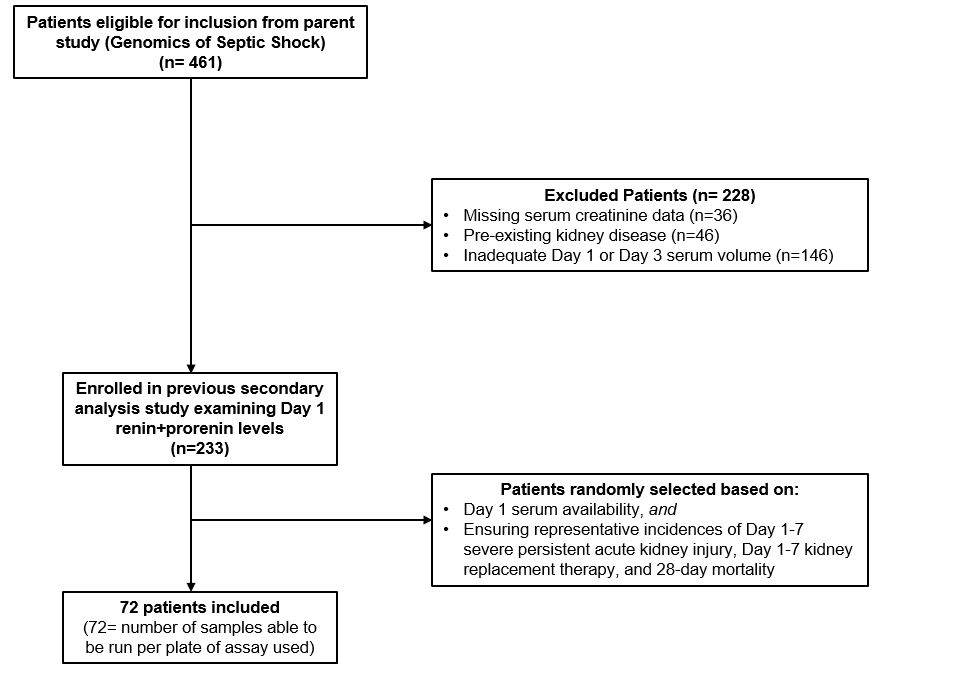


**Table S1: Patient outcomes by ACE activity and ACE concentration-derived phenotypes.**

| **Variable** | **(-) ACE Activity**  **ACE > Median** | **(-) ACE Activity**  **ACE < Median** | **(+) ACE Activity**  **ACE > Median** | **(+) ACE Activity**  **ACE < Median** | ***p*-value** |
| --- | --- | --- | --- | --- | --- |
| N (% cohort) | 24 (33.3) | 26 (36.1) | 12 (16.7) | 10 (13.9) | -- |
| Day 1-7 Severe Persistent AKI, n (%) | 13 (54.2) | 4 (15.4) | 3 (25) | 0 (0) | 0.003 |
| Day 1-7 KRT, n (%) | 8 (33.3) | 2 (7.7) | 2 (16.7) | 0 (0) | 0.04 |
| 28 Day Mortality, n (%) | 9 (37.5) | 7 (26.9) | 3 (25) | 0 (0) | 0.16 |
| Composite Outcome | 14 (58.3) | 9 (34.6) | 4 (33.3) | 0 (0) | 0.014 |
| 28 Day ICU Free Days | 6.5 (0-21) | 17.5 (0-24) | 21.5 (0.25-25) | 15 (3.75-25) | 0.40 |

Note: ACE Median= 104 ng/ml
