# Supplementary material for: Increasing angiotensin-converting enzyme concentrations and absent angiotensin-converting enzyme activity are associated with adverse kidney outcomes in pediatric septic shock
Source: Crit Care. 2023 Jun 12;27:230. doi: 10.1186/s13054-023-04518-2 (PMC10259008; doi:10.1186/s13054-023-04518-2)
Supplement: Supplementary file 2 — Additional file 2: Methods. [file 13054_2023_4518_MOESM2_ESM.docx]

**SUPPLEMENTAL METHODS:**

***Additional Original Study Details:***

The original study is a multicenter, prospective, observational cohort study of children aged 1 week to 18 years with septic shock (definition provided below) conducted rom 1/2015 to 12/2018. The original study protocol was approved by the Cincinnati Children’s Hospital Medical Center Institutional Review Board prior to patient enrollment, and includes approval for future secondary analyses of de-identified data and biospecimens. Patients from the original parent study (n=461) were excluded from the previous secondary analysis cohort if they were missing serum creatinine data for assessment of AKI (n=36), had history of pre-existing kidney disease (n=46), or did not have residual serum available for analysis (n=146) (**Additional File 1,** **Figure S1**).

All clinical and laboratory data aside from the measured RAAS components (ACE concentration, ACE activity and renin+prorenin concentrations) were measured as part of clinical care and collected for up to 7 days as part of the original study. Mortality and length of stay were tracked for 28 days after enrollment.

***Septic Shock Criteria for Original Study*** [1]***:***

1. At least 2 systemic inflammatory response syndrome (SIRS) criteria (see below) secondary to proven or suspected infection, PLUS
   - Temperature >38 or <36 degrees Celsius
   - Heart rate >90^th^ percentile for age
   - Respiratory rate >90^th^ percentile for age, or hyperventilation to PaCO2 <32 Torr
   - White blood cell count >12,000 or <4,000
2. Cultures either pending or positive, PLUS
3. Two distinct measurements of hypotension (mean arterial pressure or systolic blood pressure <3^rd^ percentile for age) after 20 ml/kg of crystalloid or colloid, PLUS
4. At least one of the following:
   - Requirement of vasoactive support, OR
   - GCS <15 (in the absence of CNS disease), OR
   - Blood lactic acid level >1.6 mmol/L, OR
   - Urine output <1 ml/kg/hr

***Serum Samples and Assay Information:***

Serum samples from Day 1 of septic shock (within 24 hours of presentation) were collected as part of the original study and stored at -80 degrees Celsius. Samples were analyzed for ACE concentrations using a human ACE Quantikine ELISA Kit (R&D Systems Inc., Minneapolis, MN, USA), and ACE activity using a high sensitivity enzymatic assay (Bulhmann Diagnostics Corp, Amherst, NH, USA). As part of a previous secondary analysis[2], serum renin concentrations were quantified using a human renin Luminex® assay (R&D Systems Inc., Minneapolis, MN, USA). This assay was selected due to small volume of residual serum available, however, may also measure prorenin, the inactive proenzyme form of renin (typically 5- to 10-fold higher than renin) [3], and thus are denoted renin+prorenin concentrations.

***Outcomes and Definitions:***

Secondary outcomes assessed included the individual components of the composite outcome (Day 1-7 severe persistent AKI, Day 1-7 KRT use, 28-day mortality), 28-day PICU-free days (calculated by subtracting the number of days in the PICU from a maximum of 28 days; patients who died in PICU before Day 28 were assigned “0”), 7-day vasoactive-free days (calculated by subtracting the number of days on vasoactives from a maximum of 7 days; those who died on vasoactives before Day 7 were assigned “0”).

**References:**

1. Wong HR, Caldwell JT, Cvijanovich NZ, Weiss SL, Fitzgerald JC, Bigham MT, et al. Prospective clinical testing and experimental validation of the Pediatric Sepsis Biomarker Risk Model. Science Translational Medicine [Internet]. 2019 [cited 2019 Nov 13];11. Available from: https://stm.sciencemag.org/content/11/518/eaax9000

2. Stanski NL, Shakked NP, Zhang B, Cvijanovich NZ, Fitzgerald JC, Jain PN, et al. Serum renin and prorenin concentrations predict severe persistent acute kidney injury and mortality in pediatric septic shock. Pediatr Nephrol [Internet]. 2023 [cited 2023 Mar 27]; Available from: https://doi.org/10.1007/s00467-023-05930-0

3. Schroten NF, Gaillard CAJM, van Veldhuisen DJ, Szymanski MK, Hillege HL, de Boer RA. New roles for renin and prorenin in heart failure and cardiorenal crosstalk. Heart Fail Rev. 2012;17:191–201.
